# *limma* powers differential expression analyses for RNA-sequencing and microarray studies

**DOI:** 10.1093/nar/gkv007

**Published:** 2015-01-20

**Authors:** Matthew E. Ritchie, Belinda Phipson, Di Wu, Yifang Hu, Charity W. Law, Wei Shi, Gordon K. Smyth

**Affiliations:** 1 Molecular Medicine Division, The Walter and Eliza Hall Institute of Medical Research, 1G Royal Parade, Parkville, Victoria 3052, Australia; 2 Department of Mathematics and Statistics, The University of Melbourne, Parkville, Victoria 3010, Australia; 3 Murdoch Childrens Research Institute, Royal Children's Hospital, 50 Flemington Road, Parkville, Victoria 3052, Australia; 4 Department of Statistics, Harvard University, 1 Oxford Street, Cambridge, MA 02138-2901, USA; 5 Bioinformatics Division, The Walter and Eliza Hall Institute of Medical Research, 1G Royal Parade, Parkville, Victoria 3052, Australia; 6 Institute of Molecular Life Sciences, University of Zurich, Winterthurerstrasse 190, Zurich 8057, Switzerland; 7 Department of Computing and Information Systems, The University of Melbourne, Parkville, Victoria 3010, Australia

## Abstract

*limma* is an R/Bioconductor software package that provides an integrated solution for analysing data from gene expression experiments. It contains rich features for handling complex experimental designs and for information borrowing to overcome the problem of small sample sizes. Over the past decade, *limma* has been a popular choice for gene discovery through differential expression analyses of microarray and high-throughput PCR data. The package contains particularly strong facilities for reading, normalizing and exploring such data. Recently, the capabilities of *limma* have been significantly expanded in two important directions. First, the package can now perform both differential expression and differential splicing analyses of RNA sequencing (RNA-seq) data. All the downstream analysis tools previously restricted to microarray data are now available for RNA-seq as well. These capabilities allow users to analyse both RNA-seq and microarray data with very similar pipelines. Second, the package is now able to go past the traditional gene-wise expression analyses in a variety of ways, analysing expression profiles in terms of co-regulated sets of genes or in terms of higher-order expression signatures. This provides enhanced possibilities for biological interpretation of gene expression differences. This article reviews the philosophy and design of the *limma* package, summarizing both new and historical features, with an emphasis on recent enhancements and features that have not been previously described.

## INTRODUCTION

Gene expression technologies are used frequently in molecular biology research to gain a snapshot of transcriptional activity in different tissues or populations of cells. These profiles are then compared to identify gene expression changes associated with a treatment condition or phenotype of interest. Gene expression studies may be randomized designed experiments in which a biological system is perturbed, for example by a gene knock-out or by applying a specified stressor. Such experiments are amongst the most powerful tools in functional genomics, providing insights into normal cellular processes as well as disease pathogenesis. Or they may be observational studies in which different phenotypes are compared, diseased and normal tissue for example or cells from different populations. Such studies are common in cancer research and in the study of cell development. In either case, the study design can range from simple two group comparisons to complex set-ups with several experimental factors varying over multiple levels. Researchers might be interested for example in whether a particular gene facilitates or blocks the action of a particular drug, in which case knock-down and wild-type samples both with and without drug treatment would be profiled. Observational studies may involve multiple batch effects and covariates that must be accounted for in the analysis.

Despite the complexity, gene expression studies often involve only a small number of biological replicates. The small but complex nature of gene expression studies poses challenging statistical problems and motivates the use of a number of specialized statistical techniques in order to get the most out of each data set. We have developed the *limma* software over the past decade to provide a framework for analysing gene expression experiments from beginning to end in a flexible and statistically rigorous way.

The *limma* package is a core component of Bioconductor, an R-based open-source software development project in statistical genomics (1,2). It has proven a popular choice for the analysis of data from experiments involving microarrays (3,4), high-throughput polymerase chain reaction (PCR) (5), protein arrays (6) and other platforms. The package is designed in such a way that, after initial pre-processing and normalization, the same analysis pipeline is used for data from all technologies.

Recently, the capabilities of *limma* have expanded significantly in two important directions. First, the package can now perform both differential expression (DE) and differential splicing analyses of RNA sequencing (RNA-seq) data (7,8). All the downstream analysis tools previously restricted to microarray data are now available for RNA-seq as well. These capabilities allow users to analyse both RNA-seq and microarray data with very similar pipelines. Second, the package is now able to go past the traditional gene-wise expression analyses in a variety of ways, analysing expression profiles in terms of co-regulated sets of genes or in terms of higher-order expression signatures (7). This provides enhanced possibilities for biological interpretation of gene expression differences.

This article reviews the philosophy and design of the *limma* package, summarizing both new and historical features, with an emphasis on recent enhancements and features that have not been previously described. The article outlines *limma*'s functionality at each of the main steps in a gene expression analysis, from data import, pre-processing, quality assessment and normalization, through to linear modelling, DE and gene signature analyses.

## MATERIALS AND METHODS

Figure 3 shows example diagnostic plots. Panel (A) shows RNA-seq data from Pickrell *et al*. (9) that has been analysed as described by Law *et al*. (10). Panels (B) and (C) display the two-colour microarray quality control data set presented by Ritchie *et al*. (11). Panel (B) displays background corrected but non-normalized intensities from one typical array. Panel (C) was generated from a subset of 30 of the control arrays after print-tip loess normalization (12).

Figure 4 shows example DE summary plots. Panels (A) and (B) were generated using the two-colour microarray data from GEO series GSE2593. Intensities were background corrected and normalized as previously described (13). Panel (A) shows a volcano plot for the comparison of samples with RUNX1 over-expressed versus wild-type samples, while panel (B) shows a Venn diagram of differentially expressed probes for each of the three over-expressed genes versus wild-type. Probes with false discovery rate less than 0.05 were considered to be differentially expressed. Panel (C) uses RNA-seq data from GEO series GSE52870. The data were analysed as described in Figure 5 of Liu *et al*. (7).

## RESULTS

### Statistical principles

**Figure 1.**
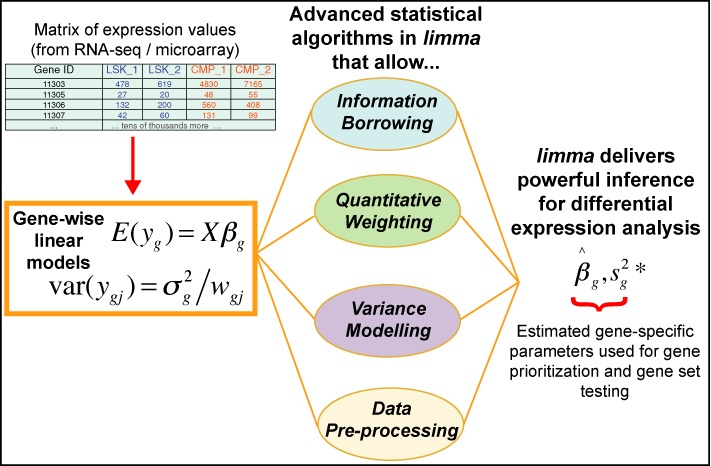
Schematic of the major components that are central to any *limma* analysis. For each gene *g*, we have a vector of gene expression values (*y*_*g*_) and a design matrix *X* that relates these values to some coefficients of interest (β_*g*_). The *limma* package includes statistical methods that (i) facilitate information borrowing using empirical Bayes methods to obtain posterior variance estimators ($s^{2*}_g$), (ii) incorporate observation weights (*w*_*gj*_ where *j* refers to sample) to allow for variations in data quality, (iii) allow variance modelling to accommodate technical or biological heterogeneity that may be present and (iv) pre-processing methods such as variance stabilization to reduce noise. These methods all help improve inference at both the gene and gene set level in small experiments.

*limma* integrates a number of statistical principles in a way that is effective for large-scale expression studies. It operates on a matrix of expression values, where each row represents a gene, or some other genomic feature relevant to the current study, and each column corresponds to an RNA sample. On the one hand, it fits a linear model to each row of data and takes advantage of the flexibility of such models in various ways, for example to handle complex experimental designs and to test very flexible hypotheses. On the other hand, it leverages the highly parallel nature of genomic data to borrow strength between the gene-wise models, allowing for different levels of variability between genes and between samples, and making statistical conclusions more reliable when the number of samples is small. All the features of the statistical models can be accessed not just for gene-wise expression analyses but also for higher level analyses of gene expression signatures. Figure 1 depicts the linear model and highlights the statistical principles employed in a typical *limma* analysis.

#### Linear models analyse complete experiments together

The hallmark of the *limma* approach is the use of linear models to analyse entire experiments as an integrated whole rather than making piece-meal comparisons between pairs of treatments. This has the effect of sharing information between samples. Analysing the data as a whole also allows us to model correlations that may exist between samples due to repeated measures or other causes. This kind of analysis would not be feasible were the data partitioned into subsets and analysed as a series of pairwise comparisons.

Linear models permit very general analyses. Researchers can adjust for the effects of multiple experimental factors or can adjust for batch effects. The linear model might include time course effects or regression splines. The linear model could even include the expression values themselves of one or more genes as covariates, allowing researchers to test for inter-gene dependencies. Linear models allow researchers to test very flexible hypotheses, not just simple comparisons between groups but also interaction effects or more complex customized comparisons.

#### Shared global parameters link gene-wise models

A separate model is fitted for each gene, but the gene-wise models can be linked by global parameters or global hyper-parameters. The use of global parameters is a simple means of sharing information between genes that can be used even for the smallest experiments, because the global parameters can be estimated from the entire data set involving all the genes at once. This strategy allows the gene-wise models to incorporate such things as correlations between duplicate probes for the same gene, or correlations between related RNA samples, or variations in quality between the RNA samples.

#### Empirical Bayes borrows information between genes

The highly parallel nature of gene expression experiments lends itself to a particular class of statistical methods, called parametric empirical Bayes, that borrow information between genes in a dynamic way (14,15). The fact that the same linear model is fitted to each gene allows us to borrow strength between genes in order to moderate the residual variances (16). The estimated variance for each gene then becomes a compromise between the gene-wise estimator, obtained from the data for that gene alone, and the global variability across all genes, estimated by pooling the ensemble of all genes. This has the effect of increasing the effective degrees of freedom with which the gene-wise variances are estimated. It was an innovation of the *limma* package to show that exact small-sample inference could be conducted using the empirical Bayes posterior variance estimators (16). This approach has proven particularly advantageous in experiments with small sample sizes, ensuring that inference is reliable and stable even when the number of replicates is small.

In recent years, the empirical Bayes procedures of *limma* have been enhanced in two important ways. First, the global variance estimate can now incorporate a mean-variance trend (10,17,18). This is important because many gene expression technologies produce data that are less reliable at lower intensities or abundances. Second, the relative weighting of the gene-wise and global variance estimators no longer needs to be the same for all genes. This allows a sophisticated robust empirical Bayes procedure in which hyper-variable genes are identified and treated separately (18,19). Both of these enhancements improve statistical power and accuracy by improving the modelling of the global characteristics of the data in a more flexible way.

#### Quantitative weights allow for unequal quality

Another unique feature of *limma* is the ability to incorporate quantitative weights into all levels of the statistical analysis, from normalization to linear modelling and gene set testing. Weights can be applied to genes or to RNA samples or to individual expression values. Weights can be used to give more emphasis to control probes during normalization, or can be used to down-weight measurements or samples that are less reliable in a gene expression analysis. The weights can be preset based on external quality information, or may be estimated from the expression data itself. The use of weights increases power to detect differentially expressed genes, and having a model based approach avoids the need for *ad hoc* decisions about which observations or samples to filter out (11).

#### RNA-seq and sequence data

All the downstream analysis features of *limma* are available for RNA-seq and other sequence count data, as well as for data from microarrays and other platforms. Traditionally, RNA-seq data require specialized software based on the negative binomial or similar distributions (20). *limma* however is able to analyse RNA-seq read counts with high precision by converting counts to the log-scale and estimating the mean-variance relationship empirically (Figure 3A). The mean-variance trend is converted by the voom function into precision weights, which are incorporated into the analysis of log-transformed RNA-seq counts using the same linear modelling commands as for microarrays. The resulting pipeline gives comparable performance to the best of the negative binomial-based software packages but with greater speed and reliability for large data sets (10,21). Additionally, and conveniently, only minimal pipeline changes are required when switching between analyses for RNA-seq and microarray experiments within *limma*. This also means that the same statistical tests with the same format of results and graphical displays are available for both data types.

#### Variance models allow for unequal variability

Expression values often show some degree of heteroscedasticity, either because there is a relationship between abundance and measurement precision, or because some treatment conditions are more heterogeneous than others. For example, tumours might be more variable than normal tissue. Concern for such effects has prompted some researchers to filter out low intensity observations or to use Welch's *t*-test for DE between two groups instead of classical pooled *t*-tests. The use of weights and the ability to model global parameters allow *limma* to incorporate unequal variances in a number of ways. One way is through estimating a mean-variance trend, which can either be incorporated into the empirical Bayes procedure as mentioned above or used to generate observation weights (10). A recent development is the ability to estimate precision weights associated with treatment groups or more generally with any given set of covariates. More generally again, the mean-variance trend can be estimated in a treatment-specific way, combining the two types of heteroscedasticity mentioned above. These approaches allow *limma* to model unequal variances even for experiments with a small number of RNA samples. Importantly, they accommodate unequal variances without compromising the linear modelling and empirical Bayes framework of the package.

#### Using sets of genes to represent higher-level expression signatures

In recent years, the linear modelling capabilities of *limma* have been extended to higher-level expression signature analyses involving co-regulated sets of genes. The idea is to use a set of genes, together with their log-fold-changes, to represent the transcriptional signature of a biological process or cell type. One way that this is done is by rotation technology, which permits statistical significance to be tested for sets of genes for any linear model contrast (55). A particular feature of rotation tests is the ability to incorporate prior information about the direction and strength with which each gene is expected to contribute to the statistical signature. In this way, *limma* provides a uniquely flexible means to relate new expression data sets to previous results collated from earlier experiments, taking into account for example the fold-change and direction of change for each gene in the earlier experiment.

A closely related statistical approach implemented in *limma* is to fit global covariance models, either to estimate correlations between genes or to estimate the relatedness between the DE profiles resulting from difference comparisons. These new analyses are described briefly later in this article.

#### Pre-processing methods preserve information

Microarray expression data are measured as intensities, which need to be background corrected and normalized before any statistical analysis can be conducted. *limma* includes a range of background correction and normalization procedures suitable for different types of DNA microarrays or protein arrays. Notable are the maximum likelihood implementation of the normal-exponential convolution model for background correction (22) and the implementation of loess curves and normalization using quantitative weights. The guiding principle in the pre-processing steps is to preserve information, avoiding missing values or inflated variances (23). Normalized intensities are offset from zero before transforming to the log-scale to avoid missing values or large variances. Offsets in a range of moderate values have been shown to achieve an effective compromise between noise and bias (24).

#### Mean-difference plots

Measuring expression in multiple RNA samples produces columns of correlated expression values, which are highly correlated because they are measured on the same set of genes or genomic features. It has long been established in the biomedical literature that the level of agreement between correlated variables can be usefully examined by plotting differences versus means. Such a plot is called a Bland–Altman plot (25) or a Tukey mean-difference plot (26). Indeed the concept of DE can be viewed as a measure of disagreement between expression measures for the same genes in different samples. Mean-difference plots were introduced to the two-colour microarray literature by Dudoit *et al*. (27) and to the single-channel literature by Bolstad *et al*. (28), who called them MA-plots. *limma* generalized the concept of an MA-plot in two ways. First, the idea was extended to apply to sets of single-channel expression values. In this case, the plot is used to compare each sample to the average of all other samples. A virtual array is constructed by averaging the log-expression value for all the samples other than the sample of interest, and then a mean-difference plot is made between the single array and the virtual array. Second, the idea was extended to apply to the fitted model objects. In this case, the plot compares the log-fold-changes for a chosen contrast versus the average log-expression values of each gene across all samples. In effect, this plots a coefficient of the linear model versus an overall mean intercept parameter. These ideas were part of the original *limma* package submitted to Bioconductor in 2003.

#### Parametric modelling versus permutation methods

It is worth mentioning what *limma* does not do, which is permutation or re-sampling-based inference. Permutation is frequently useful in large-scale studies when the aim is to compare two groups. However permutation has a number of disadvantages that make it unattractive for assessing DE in experiments with complex designs. If permutation is applied only to samples involved in two treatment conditions to be compared, then the typically small number of replicates is a severe limitation that will result in low power to detect differences. If permutation is applied to all the samples in a multi-factor experiment, then the composite null hypothesis being testing is an uninteresting one and the power to reject it may be highly dependent on the existence of DE between treatment conditions that are not of primary interest. In other words, permutation cannot be tuned to test specific null hypotheses of interest in a designed experiment. Even more importantly, permutation assumes that all samples are independent and identically distributed under the null hypothesis, and these assumptions are frequently, usually perhaps, unrealistic. In addition, permutation is potentially misleading when the samples are correlated or of unequal precision. In other words, permutation is unable to accommodate blocking structures or quality weights. In small, complex experiments, the potential compromises involved in modelling expression values using parametric distributions, which can never be perfectly correct, are outweighed by the gains in precision and accuracy by modelling the variance structure more realistically.

### Pre-processing RNA-seq and other sequencing data

**Figure 2.**
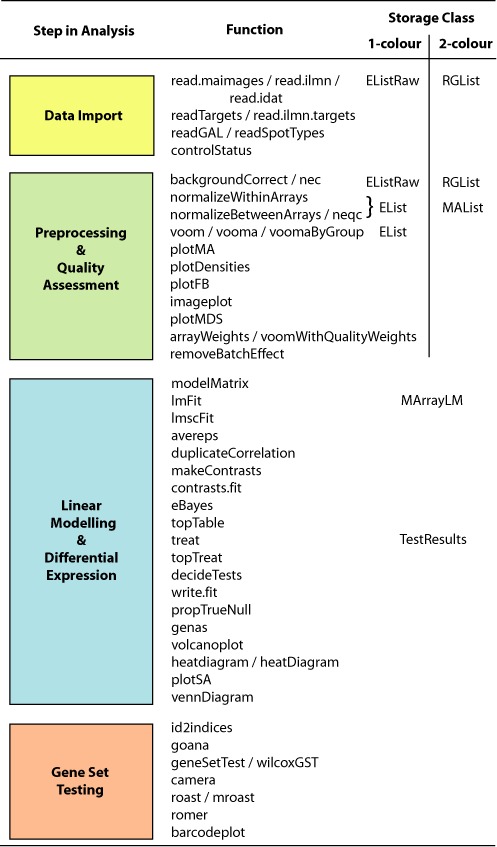
The *limma* workflow. The diagram shows the main steps in a gene expression analysis, along with individual functions that might be used and the corresponding classes used to store data or results. Online documentation pages are available both for each individual function and for each major step.

Figure 2 provides an overview of the functions available at each stage of a gene expression analysis. The first step is to import expression data into the R session.

*limma* accepts RNA-seq data in the form of a matrix of read counts, with rows for genomic features and columns for RNA samples. Alternatively it can accept a DGEList object from the *edgeR* package. The genomic regions are often genes or exons, but could in principle be any genomic feature of interest. In this article, the regions will usually be called genes for simplicity of terminology. The read counts are processed by the voom function in *limma* to convert them into log_2_ counts per million (logCPM) with associated precision weights. The logCPM values can be normalized between samples by the voom function or can be pre-normalized by adding normalization factors within *edgeR*.

Raw read counts are assembled outside *limma* using tools such as featureCounts (29), HTSeq-counts (30) or RSEM (31). The authors of this article find the Subread (32) and featureCounts pipeline particularly convenient because it is fast, accurate (8) and can be run from the R prompt using the *Rsubread* package. The data input to *limma* should be counts, rather than popular expression summaries such as reads-per-kilobase-per-million (RPKM), so that *limma* can estimate the appropriate mean-variance relationship. The voom output can be converted to RPKM values for convenience of interpretation, by subtracting log-gene-lengths, but this should be done after running voom rather than before.

After running voom, downstream analysis for RNA-seq data is the same as for any other technology. For example, RNA-seq data can be explored using boxplots or mean-difference plots, similarly to single-channel microarray data. More detail about this is given in the following sections.

### Preprocessing microarray data

#### Reading or importing data

For DNA or protein microarrays, importing expression data often involves reading output files created by an image analysis program. Alternatively, a data frame of expression values may be read from a file or data might be directly imported as an R object.

The main *limma* function to read image output files is read.maimages. This function directly supports formats written by many different image analysis programs including GenePix, Agilent Feature Extraction, ArrayVision, BlueFuse, ImaGene, QuantArray and SPOT (Table 1). It also supports the Stanford Microarray Database format. Output in other formats can be read if the appropriate column names are supplied. Two-colour and single-channel data are both supported. Illumina BeadChips need special treatment: output from Illumina's GenomeStudio can be read by read.ilmn if exported as a text file or by read.idat if in binary format.

**Table 1.** Standard microarray data formats handled by *limma*

Software	Vendor	Channels
Agilent Feature Extraction	Agilent Technologies	1/2
ArrayVision	GE Healthcare	1/2
BlueFuse	BlueGnome	1/2
GenePix	Molecular Devices	1/2
BeadScan/GenomeStudio	Illumina Inc.	1
ImaGene	BioDiscovery	1/2
QuantArray	PerkinElmer Life Sciences	1/2
ScanArray Express	PerkinElmer Life Sciences	1/2
SMD	Stanford	1/2
Spot	CSIRO	1/2

Data output by the above software can be read-in using read.maimages or read.ilmn. limma can read files in other formats, provided the user provides the names of the columns containing foreground and background intensities.

Probe annotation is read automatically if contained in the image output files, or can be read separately and added to the data object. readGAL supports the GenePix Gene Array List format. read.maimages includes the ability to generate spot quality weights according to any user-specified rule based on any information found in the image output files.

*limma* includes many possibilities for using or highlighting different types of control probes. The functions readSpotTypes and controlStatus are provided to conveniently classify probes based on text found in the input files. The status of each probe is automatically carried through to appropriate downstream functions.

The function readTargets is provided to read information about the RNA samples or *targets*. This information typically includes information about the treatment conditions and experimental design.

*limma* can accept data objects containing expression data from other Bioconductor packages. It can accept marrayNorm objects from the *marray* package, PLMset objects from the *affyPLM* package, vsn objects from the *vsn* package or objects of any class inheriting from ExpressionSet. Alternatively, expression data can be supplied as a numeric matrix. Expression values can be image intensities or normalized log-expression values.

#### Background correction

When array images are read, it is usual to read both foreground and background intensities for each probe. The background intensities can be used to derive an estimate of the ambient intensity affecting each probe. Removing this non-specific signal from the foreground intensity of each probe is called background correction and it is typically the first step in processing microarray images. Simply subtracting background from foreground intensities is too heavy-handed (23). The *limma* backgroundCorrect function offers a range of more sophisticated alternatives, most unique to the package. These include a method based on a convolution of normal distributions (33) and a normal-exponential (*normexp*) convolution (23) with different options for parameter estimation (22). The plotFB function plots foreground against background intensities for each array and is useful for choosing an appropriate correction method.

Illumina arrays again benefit from special treatment. The nec function implements normexp background correction for Illumina BeadChips making special use of the control probes that are specific to these arrays (24).

The propexpr function compares intensities to those of negative control probes to estimate the total proportion of probes on each array that correspond to expressed genes (34). This provides an estimate of the size of the transcriptome in each sample and is useful for deciding how many probes to filter from downstream analyses.

#### Normalization

Before meaningful comparisons can be made between treatment conditions in a designed experiment, it is critical that the expression values are normalized so that all the samples are as far as possible on the same measurement scale. The purpose of normalization is to remove systematic effects due to technical differences between the assays unassociated with the biological differences of interest. Different technology platforms introduce different biases and so require different normalization methods. The normalizeWithinArrays function normalizes data from two-colour microarrays by aligning the two channels for each array. A popular method is to remove intensity-dependent dye-biases and spatial artefacts from *M*-values (log-intensity ratios) using locally weighted regression (loess) (35). The normalizeBetweenArrays function aligns expression values between samples for one-colour microarrays and other single channel platforms using methods such as quantile normalization or cyclic loess (28). Both functions provide a range of different normalization methods suitable for different platforms. normalizeBetweenArrays also implements separate channel normalization methods for two-colour arrays (36,37). *limma* is the only software to allow the use of quantitative weights in loess normalization (38), giving it the ability to downweigh less reliable probes or to give higher priority to control probes or house-keeping genes. The latter ability has been exploited for normalizing assays when the proportion of differentially expressed genes may be high, for example boutique arrays (39), miRNA arrays (40), PCR arrays, protein arrays or protein mass spectrometry. Other enhancements include the ability to replace the loess curve with a spline curve that has high robustness breakdown properties, and the ability to apply empirical Bayes moderation to the spline curves for multiple regions within the same array (robust-spline normalization).

The neqc function implements quantile normalization for Illumina BeadChips making special use of the control probes specific to these arrays (24).

All the between-arrays normalization methods are accessible for RNA-seq data from within the voom function. Alternatively, voom has the ability to respect normalization factors computed outside of *limma* by methods such as trimmed mean of *M*-values (41) or conditional quantile normalization (42).

### Graphical exploration of data quality

**Figure 3.**
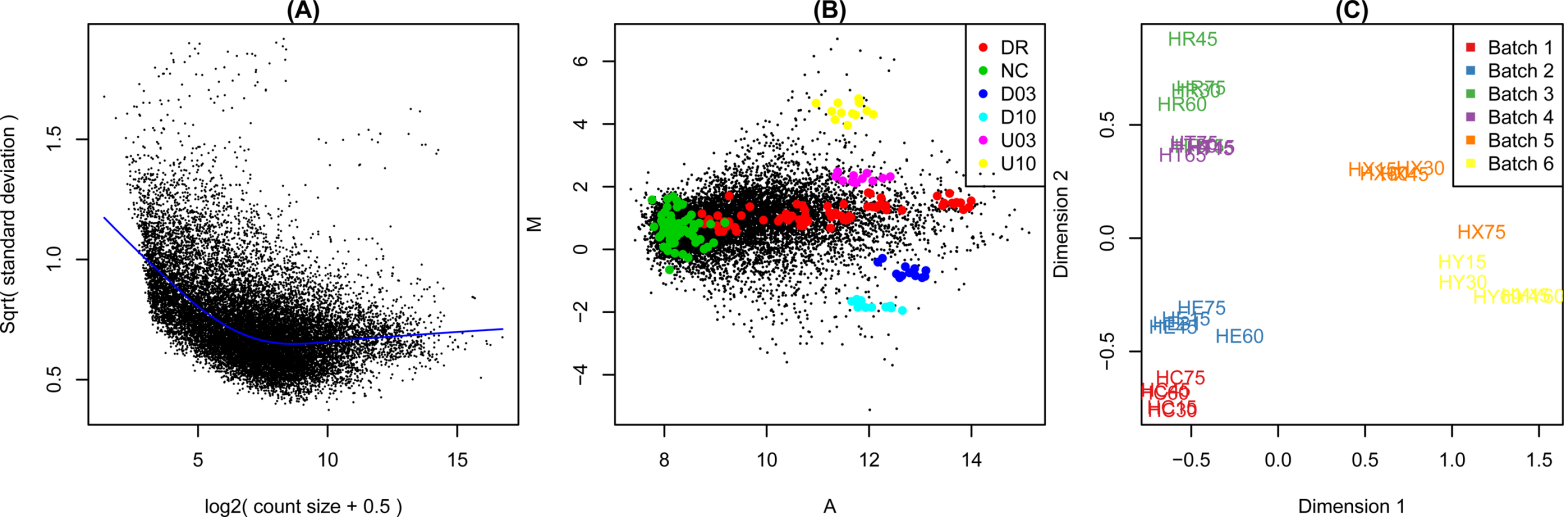
Example diagnostic plots produced by *limma*. (**A**) Plot of variability versus count size for RNA-seq data, generated by voom with plot=TRUE. This plot shows that technical variability decreases with count size. Total variability asymptotes to biological variability as count sizes increases. (**B**) Mean-difference plot produced by the plotMA function for a two-colour microarray. The plot highlights negative (NC), constant (DR) and differentially expressed (D03, D10, U03, U10) spike-in controls. Regular probes are non-highlighted. (**C**) Multidimensional scaling (MDS) plot of a set of 30 microarrays, generated by plotMDS. All arrays are biologically identical and the plot reveals strong batch effects. Distances represent leading log2-fold changes between samples.

Diagnostic plots allow the user to visually inspect data from a designed experiment in order to identify potential quality problems, such as degraded samples, or problems that arise due to array handling or sample processing. Such displays may also reveal systematic biases that should be removed prior to downstream analysis. Figure 3 presents examples from three different plotting functions.

Plots for individual arrays include the foreground–background plots mentioned above (plotFG), image plots that can reveal inconsistencies across the array surface (

imageplot) and mean-difference plots that show intensity-dependent trends in the log-ratios of two-colour arrays (plotMA, Figure 3B). The plotMA function can show similar plots for single channel data. In this case, the mean-difference plot is constructed by comparing the log-expression values for that sample compared with the mean of all other samples. The plotMA function makes it simple to highlight particular subsets of probes or genes, for example control probes. Control probes are automatically highlighted if they have previously been identified using controlStatus (Figure 3B).

The distribution of expression values can be compared between samples using box plots or density plots (plotDensities). The latter is particularly useful when considering separate channel analyses of two-colour arrays.

### Finding differentially expressed genes

#### Exploration of sample relationships

After the pre-processing steps described above, the next major analysis stage is to identify differentially expressed genes. It is advisable to begin the DE analysis with a plot that visualizes the relative differences in transcriptional profile between the samples. The plotMDS function uses multi-dimensional scaling to plot differences in expression profiles between different samples (Figure 3C). Distances between samples on the plot represent ‘leading fold change’, which is defined as the root-mean-square average of the log-fold-changes for the genes best distinguishing each pair of samples. This provides a type of unsupervised clustering of the samples. It is useful for examining how different are the profiles produced by different experimental factors and for identifying unexpected patterns, such as batch effects, that should be adjusted for during the linear model analysis. This helps guide the construction of the design matrix used for the linear modelling below.

The plotRLDF function provides a supervised plot of the samples that shows whether the expression data can distinguish a set of known groups. The function implements a regularized version of linear discriminant functions.

The removeBatchEffect function can be used to remove systematic variation due to batches or other covariates prior to plotting the data so that the effect of treatments can be better seen.

#### Linear modelling

The core component of the *limma* package is the ability to fit gene-wise linear models to gene expression data in order to assess DE (16). The basic idea is to estimate log-ratios (for two-channel data) or log-intensities (for single-channel data) between two or more target RNA samples simultaneously.

Each analysis begins with a matrix of expression levels, with probes/genes/exons in the rows and different samples (biological/technical replicates) in the columns. The linear modelling is performed in a row-wise fashion, with regression coefficients and standard errors either directly estimating the comparisons of interest or via contrasts. Test-statistics are obtained for gene ranking that can be further summarized at the gene set level to perform gene signature/pathway-level ranking.

The flexibility of the linear modelling approach allows almost any experimental design to be handled. Experiments with two or more groups, factorial and time-course designs, and internal controls such as dye-swaps can all be modelled and summarized using the lmFit function. Where appropriate, nuisance variables such as batch and dye effects can also be modelled. Models can be fit robustly or by least squares. Once a linear model is fitted, the makeContrasts function can be used to form a contrast matrix. The fitted model object and contrast matrix are used by contrasts.fit to compute log_2_-fold-changes and *t*-statistics for the contrasts of interest. This allows all possible pairwise comparisons between treatments to be made.

The plotSA function provides a useful diagnostic plot of the linear model fit, plotting gene-wise residual standard deviations against average log-expression. This allows mean-variance trends to be readily identified, should they exist.

#### Quality weights and heteroscedasticity

*limma* is the only package that allows variations in quality to be handled in a graduated way via quantitative weights. Both observation-level (2,10,43) and sample-specific weights (11) can be used in an analysis. For microarray data, the arrayWeights function estimates relative array variances, which are converted to weights that can be used in the linear model analysis to down-weight observations from less reliable arrays. Probe and array weights can be easily combined by multiplying them together and, when used appropriately, have been demonstrated to increase power to detect DE (11,43).

For RNA-seq data, the voomWithQualityWeights function combines observation-level and sample-specific weights for use in the subsequent linear modelling.

#### Blocking and random effects

*limma* includes a unique strategy for incorporating the fact that observations or samples may be correlated. The strategy is similar to fitting a random effects model, with the difference that all genes are constrained to share the same intrablock correlation. The duplicateCorrelation function is used to estimate the consensus correlation. The correlation structure is then incorporated into the linear model fit and hence into all tests for DE. Originally the idea was used to estimate the correlation between replicate copies of the same probe on a microarray (44). The correlation strategy preserves more information than simply averaging the replicate probe copies. More generally, the same idea is also used to model the correlation between related RNA samples, for example repeated measures on the same individual or RNA samples collected at the same time.

#### Separate channel analysis of two-colour microarrays

Two-colour microarrays are traditionally analysed in terms of log-ratios between the two channels hybridized to each probe. *limma* also provides the possibility of analysing two-colour microarrays as if they were single channel microarrays with two separate samples hybridized to each physical array. This provides a very powerful type of analysis in which intensities can be directly compared between microarrays. The pairing of the red and green channels from each array is kept track of by estimating the correlation between the two channels hybridized to each probe (37). This type of separate channel analysis uses the intraspotCorrelation and lmscFit functions.

All the linear model fits, whether using lmFit or lmscFit, produce a fitted model object with the same structure. The same fitted model applies regardless of whether correlations have been estimated, whether robust regression or least squares has been used, or whether quality weights have been included. This consistency allows the same framework for DE to be used for all experimental designs and platform technologies.

#### Testing for DE

An empirical Bayes framework to borrow information between genes when estimating the variances is implemented in the eBayes function. Gene-wise variances are squeezed towards the common or trended variance, which reduces the number of false positives for genes with very small variances and improves power to detect DE for genes with larger variances. *limma* includes a robustified shrinkage strategy that allows for gene-wise shrinkage factors to be estimated (18). This ensures unusually large variances are not squeezed too heavily, reducing the chance that they will appear statistically significant, while more consistently expressed genes are squeezed more severely towards the common variance. This robust strategy offers the benefits of shrinkage to the majority of the genes, whilst negating the effects of outliers.

For each coefficient in the linear model or contrast, empirical Bayes moderated *t*-statistics and their associated *P*-values are generally used to assess the significance of the observed expression changes. *T*-statistics can also be translated into Bayesian log-odds of DE. Moderated *F*-statistics that combine the *t*-statistics for all contrasts into an overall test of significance for each gene can also be used.

When one has a particular cut-off for log-fold-change in mind, the treat function can be used to test whether the log_2_-fold-change is greater than a threshold rather than merely different to zero (45). This can be effective for prioritizing results that are biologically as well as statistically significant.

*limma* provides a number of options to adjust tests for multiple testing. Users can control either the family-wise type I error rate or the false discovery rate (46). As well as the usual control for multiple testing across multiple genes, *limma* is the only software package to provide methods for error rate control across multiple contrasts and genes simultaneously. For individual tests, multiple testing can be applied using the topTable function. The decideTests function gives access to the full range of options.

**Figure 4.**
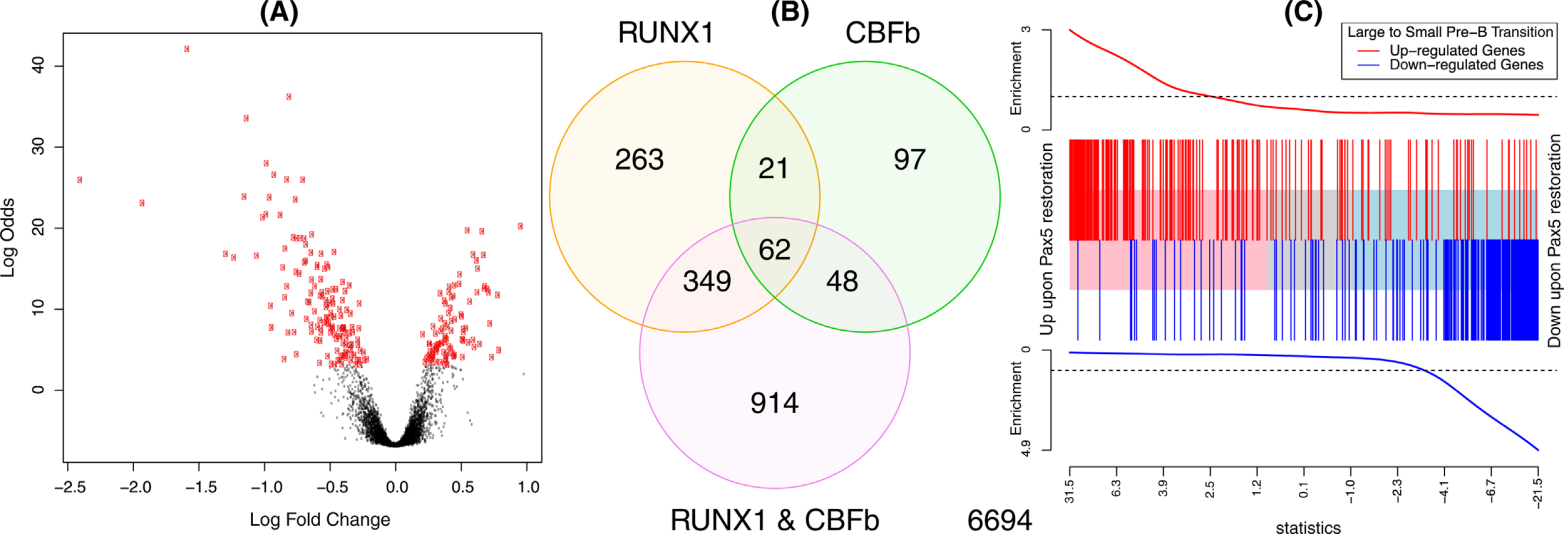
Example plots displaying results from DE and gene set analyses. (**A**) Volcano plot showing fold changes and posterior odds of DE for a particular comparison (RUNX1 over-expression versus wild-type in this case), generated by volcanoplot. Probes with *P* < 0.00001 are highlighted in red. (**B**) Venn diagram showing overlap in the number of DE genes for three comparisons from the same study as (A), generated by the vennDiagram function. (**C**) Gene set enrichment plot produced by barcodeplot. The central bar orders differentially expressed genes by significance from up to down upon Pax5 restoration in an RNA-seq experiment (7). The vertical bars mark genes that are induced (red) or repressed (blue) upon the transition from large cycling pre-B cells to small resting pre-B cells during normal B cell development according to the published literature (47). The plot shows a strong positive concordance between Pax5 restoration and the large to small cell transition. The roast function can be used to assign statistical significance to this correlation.

To visualize the results of a DE analysis for single or multiple contrasts, *limma* provides a number of plotting options. Figure 4 shows three such displays: a volcano plot showing the DE results from a single condition, a Venn diagram showing the number of differentially expressed genes in multiple experimental conditions and a barcode enrichment plot highlighting a particular gene signature in a DE analysis ranked by moderated *t*-statistics.

Another useful plot is produced by plotMA, which plots estimated log-fold-changes against mean log-expression for each gene. This allows the magnitude of changes to be visualized in the context of overall expression level; see for example Figure 5C of Liu *et al*. (7).

#### Testing for differential splicing

The linear model framework of *limma* is extended to test very easily for differential splicing events when exon-level expression data are available. The data can be either from an exon microarray or from RNA-seq data summarized at the exon level. In either case, the approach is based on fitting linear models to the exon-level expression data. The approach can relate differential exon usage to continuous as well as categorical predictors or to any contrast in a linear model. The test is conducted by the diffSplice function and results are displayed using plotSplice and topSplice. The plotExons function is also useful for exploring exon expression for individual genes. This approach is considerably faster than alternative approaches to differential splicing, making large-scale surveys of differential exon usage feasible.

### Higher-level analyses

Linear modelling of gene expression data provides the ideal platform from which to attack larger functional genomic questions related to gene-wise independence or interaction and the decomposition of gene signatures into distinct molecular pathways. This section describes higher-level analyses involving multiple genes.

#### Estimating the proportion of true null hypotheses

First we consider DE from a genomic point of view. The DE analyses described so far identify individual differentially expressed genes according to an FDR criterion. However, for most studies, there are likely to be false negatives: truly differentially expressed genes that are not detected as differentially expressed because the study did not have enough statistical power to identify them with confidence.

The propTrueNull function estimates the number of truly differentially expressed genes that remain to be identified. Mathematically, it estimates the proportion of true null hypotheses in a collection of hypothesis tests given a vector of *P*-values. In a gene expression study, it estimates the proportion of non-differentially expressed genes, out of all tested, for any contrast in the linear model. The function implements a number of different methods for estimating the proportion of true nulls, ranging from quick and simple to more computationally demanding. The default is based on averaging local false discovery rates across the *P*-values (19). Other methods are the histogram method of (48,49), the convex decreasing density estimate of (50) and a very simple estimate based on averaging the *P*-values.

#### Genuine association of gene expression profiles

Gene expression experiments typically involve a number of different treatment conditions. A question that often arises is this: to what extent do two different treatments produce similar or different expression profiles? One way to address this question is to count the overlap in differentially expressed genes from the two treatments, as in Figure 4B. This approach however is often too crude. It is very sensitive to the significance cut-off used to identify differentially expressed genes, has little chance of success in situations where power to detect differentially expressed genes is relatively low and is subject to technical biases when both treatments are compared back to the same control samples. The genas function (19) addresses these problems. It tests whether two different contrasts in a linear model affect the same genes in similar or different ways, adjusting for biases, without needing to apply a significance cut-off for assessing DE. More technically, it estimates the true biological correlation between the log_2_-fold-changes of two different contrasts. By biological correlation, we mean the correlation that would exist between log-fold-changes if they could be measured perfectly without any statistical error. genas is based on a bivariate generalization of the empirical Bayes model that is used to assess DE in *limma*. This method is particularly powerful for gaining insight into commonly affected gene pathways when the changes are small but consistent. For instance, applying genas to a microarray study looking at the relationship between polycomb repressor complex (PRC) 1 and PRC2 facilitated the discovery of the opposing roles of these two complexes (51). This relationship would have been missed if the analysis had been restricted to the statistically significant genes from each contrast alone.

#### Gene set testing

Gene set analyses assess the overall significance of a set of co-regulated genes. Each gene set is chosen to represent a particular molecular pathway or some other biological process of interest. Gene sets are defined by gene annotation external to the current expression study, for example from Gene Ontology (GO) database (52) or from previous expression studies. For gene sets defined by previous studies, the genes may optionally be annotated with the direction and magnitude of expression changes in the earlier experiment. In this way, a gene set may contain genes both positively and negatively associated with the molecular pathway that it represents.

*limma* contains a range of options for gene set testing via the goana, geneSetTest, camera, roast and romer functions. The goana function provides a traditional GO overlap analysis but with the added ability to adjust for gene length or abundance biases in RNA-seq DE detection. goana uses generalized hypergeometric tests to test for enrichment of GO terms in the list of differentially expressed genes (53). It operates directly on the fitted model object and extracts differentially expressed genes automatically.

Unlike goana, the other gene set options do not require a significance threshold to be applied to identify differentially expressed genes. The simplest approach is implemented in the geneSetTest and wilcoxGST functions, which perform rank-based tests (13). These tests assess whether the specified set of genes is more highly ranked in an ordered list of all genes than would be expected by chance. These tests have been found to give an effective ranking of biologically significant pathways (54), but they implicitly assume that the expression level of each gene is conditionally independent of other genes and hence give optimistic *P*-values (55).

More sophisticated competitive tests that take into account dependence between the genes in the linear modelling framework are implemented in the camera function (56). Camera computes a variance inflation factor from the inter-gene correlation and uses this to adjust the variance of the summary statistics. This avoids optimistic *P*-values in the test results, but also reduces statistical power. Camera has been used successfully in a number of biomedical projects (57).

The roast and mroast functions implement a self-contained test of whether any worthwhile subset of the specified set of genes is differentially expressed (55). These functions use rotation tests, a specialized simulation technique for multivariate normal models (58). Rotation can be viewed as a smoothed version of permutation that is suitable for linear models. Rotation occurs only in the residual space of the linear model in such a way that any coefficients in the linear model other than the contrast being tested are held constant (55,58). Roast can provide good statistical power in small complex gene expression experiments. Of all gene set tests, roast has the unique ability to take into account directional annotation information about genes in the set. It is able to accommodate genes both positively and negatively associated with a specified pathway, as well as the magnitude of change of each gene. It is therefore especially useful for finding similarities in gene expression patterns between different expression studies (57,59–61) (Figure 4C). Other potential applications for roast include those where the set might not be made up of genes, for example exon-level expression analyses to test whether any exon of a given gene is differentially expressed.

Gene set enrichment analysis (GSEA) is an approach that correlates a large database of co-regulated gene sets with respect to a microarray or RNA-seq data set (62,63). GSEA is a hybrid approach: it is competitive in that different sets are pitted against one another, but significance is evaluated by permutation of sample labels. The romer function in *limma* implements a GSEA approach that is based on rotation instead of permutation. Like camera and mroast, it can be used with a battery of gene sets and with any linear model. The *limma* authors maintain mouse and human versions of the Molecular Signatures Database collections (64) in R binary format that can be conveniently used with camera, mroast or romer (http://bioinf.wehi.edu.au/software/MSigDB).

The use of gene sets require that gene symbols and annotation be matched between different databases and studies. Gene symbols change over time, so *limma* includes the alias2Symbol and alias2SymbolTable functions to map gene symbol aliases to current official gene symbols. The id2indices function matches gene identifiers in a collection of gene sets to those in the expression matrix, in a format suitable for input into camera, mroast or romer.

A gene set test can be visualized using the barcodeplot function (Figure 4C). Genes are ranked according to their DE results in the current study, then genes from the *a priori* set are highlighted by vertical bars, with a smoother line showing the relative enrichment of the gene set amongst high and low ranked genes. Barcodeplot is similar to the set location plot introduced by Subramanian *et al*. (63), with a number of enhancements, especially the ability to incorporate genes with positive and negative prior directions. Barcodeplot can optionally display varying weights for different genes, for example log-fold-changes from a previous experiment.

### User-interface

#### Object-oriented programming

A simple but appropriate object-oriented paradigm provides users with a consistent analysis interface that is very easy from a user point of view. Many *limma* functions are generic or operate appropriately on objects of different classes. *limma* defines a number of classes that have been tailored to handle both microarray and RNA-seq data. The philosophy has been to define simple list-based data objects that can be easily explored and manipulated by users, in the same style as familiar, long-standing core functions in R such as lm and glm.

For raw intensity data, the classes ‘RGList’ and ‘EListRaw’ are used to store two-colour and single-channel data, respectively. These objects are often created using the function read.maimages and contain the raw values from the image analysis output files along with probe annotation information.

Normalized data are stored in ‘MAList’ or ‘EList’ objects. Normalized two-colour data are converted from red and green intensities, *R* and *G*, into *M* and *A*-values, which hold the log-ratio and average log-intensity values for each spot. Single channel data are background corrected and log_2_ transformed and stored in an ‘EList’ object. For RNA-seq data, the voom transformed matrix of gene/exon counts is also stored in an ‘EList’ object.

The next major classes store output from a DE analysis. ‘MArrayLM’ objects store the result of fitting gene-wise linear models to the normalized intensities or log-ratios. Objects of this class are created by the lmFit and eBayes functions. After running decideTests, an object of class ‘TestResults’ stores the results of testing a set of contrasts equal to zero for each probe/gene.

All of these data classes obey many analogies with matrices. In the case of ‘RGList’, ‘MAList’, ‘EListRaw’ and ‘EList’, rows correspond to probes/genes and columns to different samples. In the case of ‘MarrayLM’ and ‘TestResults’ rows correspond to unique probes/genes and the columns to linear model coefficients or contrasts. The standard R functions summary, dim, length, ncol, nrow, dimnames, rownames, colnames have methods for each of these classes. Objects of any of these classes may also be subsetted. Multiple data objects may be combined by rows to add extra probes, or by columns to add extra arrays.

Furthermore all of these classes may be coerced to be of class matrix using as.matrix, although this entails loss of information. Fitted model objects of class ‘MArrayLM’ can be coerced to class data.frame using as.data.frame in R. The first five classes belong to the virtual class ‘LargeDataObject’ for which a show method is defined to display the leading rows of a large vector, matrix or data.frame.

#### Computational efficiency

The *limma* package is implemented primarily in R (65) and includes some C code to speed up computationally intensive steps. At every stage, effort has been expended to achieve high numerical reliability and efficiency. The memory requirements are linear in the number of genes and the number of samples. Most estimation procedures finish in a few seconds on a standard desktop computer and virtually all in less than a minute.

#### Availability

The *limma* software is freely available online as part of the Bioconductor project (http://www.bioconductor.org). More than 120 other Bioconductor packages make use of *limma* (as of March 2014). The *limma* package is used as a building block or as the underlying computational engine by a number of software projects designed to provide user-interfaces for gene expression data analysis including *limmaGUI* (66), *affylmGUI* (67), WebArray (68), RACE (69), CarmaWEB (70), Goulphar (71), MAGMA (72), Asterias (73), GenePattern (74), GEO2R (http://www.ncbi.nlm.nih.gov/geo/geo2r), the EBI expression atlas (75), Guide (76) and Degust (http://www.vicbioinformatics.com/degust).

#### Documentation

The *limma* package provides three levels of documentation. First, each function has its own documentation page that concisely but completely specifies the input data, options and output format of the function. Similarly, each data class has a documentation page explaining all the required and optional components of objects of that class. Care is taken to adhere to the same standards and style that users will be familiar with from help pages in the base R packages.

Second, a series of more general subject help pages serve to link together functions and classes that are used for related purposes. The subject pages cover the topics of (i) introduction, (ii) classes, (iii) reading data, (iv) background correction, (v) normalization, (vi) linear models, (vii) individual channel analysis of two-colour data, (viii) hypothesis testing for linear models, (ix) diagnostics and quality assessment, (x) gene set tests and (xi) RNA-seq.

Third, the package comes with an extensive user's guide of over 120 pages, available from the drop-down menu in Windows or alternatively launched by the limmaUsersGuide command. The user's guide gives detailed advice on how to analyse a variety of common study designs. It also includes 10 fully worked case studies for which full data and code are provided.

Users who need more help or advice are invited to post questions to the Bioconductor support site (https://support.bioconductor.org). Questions are usually answered promptly, either by the authors or by other members of the Bioconductor community. The support site archives answers to many common questions, including many queries about experimental design and setting up appropriate design matrices.

## DISCUSSION

This article has summarized the current features of the widely used, open source *limma* package for gene expression analysis. This software provides an integrated data analysis solution, using advanced computational algorithms to deliver reliable performance on large data sets and object-oriented ideas to represent expression data and simplify the user interface. New functionality is continually being added as model refinements and new use cases arise.

Although originally developed with microarray data in mind, the development of the voom methodology unlocks the majority of analysis methods for use on RNA-seq data, such as random effects modelling and gene set testing. As with any data analysis problem, the appropriate combination of methods to use will depend upon the biological question, platform used (microarray/RNA-seq) and experimental design.

Being R-based, reports of *limma* analyses can be compiled using Sweave (77) or *knitr* (78) and provided along with the raw data in a compendium to promote reproducible research in genomics (79).

Applications of *limma*'s linear modelling strategy beyond the intended analysis of gene expression data have been made in a variety of applications, including the analysis of data from Nuclear Magnetic Resonance spectroscopy, PCR (including Nanostring), quantitative proteomics (80), DNA methylation arrays and comparative ChIP-seq (81).

As the cost of collecting genome-wide profiles continues to fall, we expect the popularity of this approach to continue to grow, with new applications in the analysis of single cell gene expression data, CRISPR/Cas9 knock-out screens and methylation analysis (82).
